# Mitochondrial Malfunctioning, Proteasome Arrest and Apoptosis in Cancer Cells by Focused Intracellular Generation of Oxygen Radicals

**DOI:** 10.3390/ijms160920375

**Published:** 2015-08-28

**Authors:** Ilaria Postiglione, Angela Chiaviello, Federica Barra, Emanuela Roscetto, Amata A. Soriano, Maria Rosaria Catania, Giuseppe Palumbo, Giovanna Maria Pierantoni

**Affiliations:** 1 Department of Molecular Medicine and Medical Biotechnology, University of Naples Federico II, Naples 80131, Italy; E-Mails: ilariapostiglione@gmail.com (I.P.); angelachiaviello@libero.it (A.C.); federica.barra@unina.it (F.B.); emanuelaroscetto@gmail.com (E.R.); amata.soriano@libero.it (A.A.S.); mariarosaria.catania@unina.it (M.R.C.); giovannamaria.pierantoni@unina.it (G.M.P.); 2 Institute of Experimental Endocrinology and Oncology (IEOS), National Research Council (CNR), Naples 80131, Italy

**Keywords:** photodynamic therapy, Photofrin, Bortezomib, combination therapy, proteasome

## Abstract

Photofrin/photodynamic therapy (PDT) at sub-lethal doses induced a transient stall in proteasome activity in surviving A549 (p53^+/+^) and H1299 (p53^−/−^) cells as indicated by the time-dependent decline/recovery of chymotrypsin-like activity. Indeed, within 3 h of incubation, Photofrin invaded the cytoplasm and localized preferentially within the mitochondria. Its light activation determined a decrease in mitochondrial membrane potential and a reversible arrest in proteasomal activity. A similar result is obtained by treating cells with Antimycin and Rotenone, indicating, as a common denominator of this effect, the ATP decrease. Both inhibitors, however, were more toxic to cells as the recovery of proteasomal activity was incomplete. We evaluated whether combining PDT (which is a treatment for killing tumor cells, *per se*, and inducing proteasome arrest in the surviving ones) with Bortezomib doses capable of sustaining the stall would protract the arrest with sufficient time to induce apoptosis in remaining cells. The evaluation of the mitochondrial membrane depolarization, residual proteasome and mitochondrial enzymatic activities, colony-forming capabilities, and changes in protein expression profiles in A549 and H1299 cells under a combined therapeutic regimen gave results consistent with our hypothesis.

## 1. Introduction

It has already been a long time since photodynamic therapy (PDT) has turned 100 years old, but it is only a few decades since it has been adopted as a clinical therapeutic approach, mostly because some photosensitizers have been approved for clinical use. Indeed, PDT effectively reduces the number of tumor cells [1], but is a common result that it is often unable to completely eradicate them. It is known that the principal cytotoxic effect of PDT is mediated by the production of reactive oxygen species that directly damage sub-cellular components including membranes and proteins. Because of the limited migration of oxygen radicals from the location of their formation, sites of initial cell or tissue damage are closely related to the docking place of the sensitizer [2]. Indeed, sensitizers can accumulate in different cellular compartments depending on their physico-chemical nature and specific cell/tissue. Several studies have demonstrated that the widely used photosensitizer Photofrin accumulates in the various cellular compartments due to its concentration and its incubation time [3]. In any case, the preferential sites appear to be represented by the mitochondria and endoplasmic reticulum [4].

To date, PDT is generally acknowledged as a potential palliative therapy, and as a primary therapy only in select conditions. In fact, PDT is widely applied in dermatology and is very effective [5]. Among the principal reasons of the partial efficacy of PDT, we find the limited penetration depth of visible light in thick tissues, the non-homogenous distribution of the photosensitizer within the tumor [6], and the oxygen shortage within the tissue as a result of the photochemical consumption of oxygen during the photodynamic process [7]. While attempts of overcoming those difficulties are currently undertaken by many, it is generally recognized that a real step forward can be made by a combination approach that can improve the therapeutic outcome by eradicting the surviving tumor cells [8]. Indeed, among the possibilities of improving PDT efficacy by combining it with chemotherapy, radiotherapy, and other treatments, it is possible to include a variety of drugs that target molecules involved in physiological protein degradation processes at the ubiquitin-proteasome pathway. This system strictly controls the degradation of most cellular proteins (~80%) in eukaryotes [9] and plays a pivotal role in the degradation of unfolded proteins in response to oxidative damages [10].

The present study, performed on the human-derived lung adenocarcinoma H1299 and A549 cell lines, describes the detrimental effects exerted by PDT in combination with Bortezomib, proves the *in vitro* efficacy of this treatment, and hypothesizes the mechanism that, under combination regimens, may force residual cells to apoptosis.

## 2. Results and Discussion

### 2.1. Subcellular Localization of Photofrin

**Figure 1:**
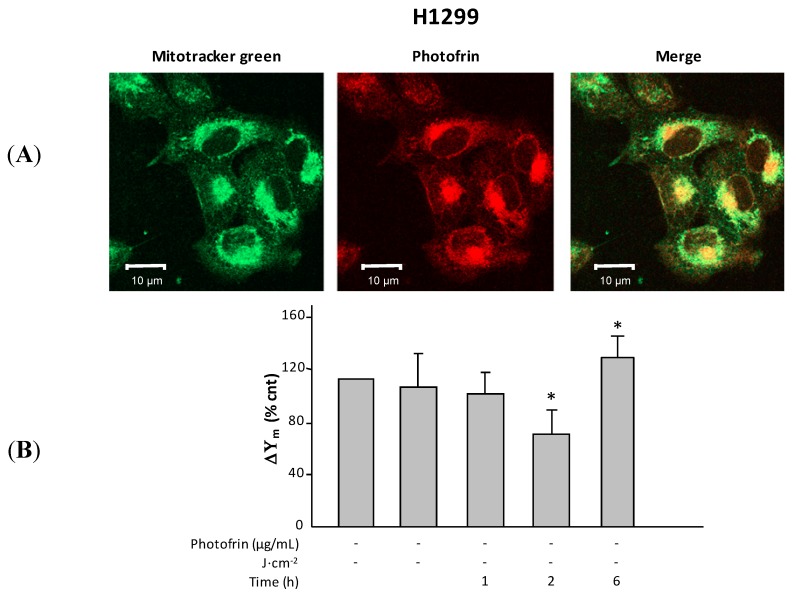
(**A**) Images from confocal microscope. **Left panel**, Mitotracker staining (green); **middle panel**, Photofrin (2.5 μg/mL for 16 h) direct emission (red); and **right panel**, merge image (yellow); (**B**) The mitochondrial membrane potential (ΔΨm) change with time after irradiation (0.54 J·cm^−2^). Untreated cells were used as controls. Note the recovery at 6 h. * *p* < 0.05.

Previous observations [11] reported that the incubation of HeLa cells with Photofrin for long intervals determines an initial accumulation of the photosensitizer at the level of the cytoplasmic membrane and, additionally, of mitochondria. The Photofrin route within cells can be easily monitored by confocal microscopy and the photosensitizer, excited at 395 nm, emits an intense red light [12]; we made timed observations of H1299 and A549 cells during the 16 hours of incubation with Photofrin (2.5 μg/mL) and noticed during the first hours that the red fluorescence was mainly concentrated along the cell membrane while, later, red fluorescence flooded the cytoplasm (not shown). Further staining of the cells with Mitotracker green, which appears to selectively localize to mitochondria regardless of their membrane potential [13], indicated its co-localization with Photofrin. This finding demonstrates that under the indicated experimental conditions, Photofrin recognizes mitochondria as a preferential localization even in H1299 and A549 cells. However, if the observed depolarization is mainly triggered by oxygen radicals generated within the mitochondria, it is also possible that it is caused by similar damage of other cellular structures, such as the Golgi apparatus. The data shown in Figure 1A are relative to the indicated conditions and refer to H1299 cells alone.

### 2.2. Photodynamic Therapy (PDT)-Induced Radical Oxygen Species (ROS) Generation Affects Mitochondrial Membrane Potential (ΔΨm)

The generation of a proton gradient across the inner mitochondrial membrane is an essential energy conservation event that combines carbohydrates and lipid oxidations to the production of ATP. Several studies have established that the mitochondrial membrane potential (ΔΨm) and, to a lesser extent, the chemical gradient for protons contribute independently to the proton-motive force that drives the synthesis of ATP [14]. The preservation and support of membrane potential is essential for the mitochondria functioning and any alteration induced by whatever detrimental cause may impair their activity. Among the various causes, an overload of intracellular radical oxygen species (ROS) must be considered [15]. In this regard, we know that PDT kills cells though local and intense formation of ROS [3] and this also holds true for H1299 and A549 cells [16]. Indeed, mitochondria represent an easy target for ROS. Consequently, we investigated where Photofrin/PDT was responsible for mitochondria dysfunction in H1299 and A549 cells. In this regard, using the MitoPTm TMRE (tetramethylrhodamine methyl ester perchlorate) kit (Immunochemistry Technologies, Bloomington, MN, USA), we observed that mitochondrial membrane potential decreases within 2 h in both cell lines (Figure 1B, above) upon irradiation. We have also observed that at longer time intervals (≈6 h post PDT), the ΔΨm improves, suggesting that at moderate light fluence (*i.e.*, 0.54 J·cm^−2^, which is proficient in killing ~50% of all cells), the mitochondrial impairment is not irreversible. The increase of membrane potential observed at six hours may represent a rebound effect determined by the moderate photodynamic treatment. Notably, Photofrin is not directly involved in this process since its administration without exposure to light does not alter mitochondrial membrane potential.

### 2.3. Mitochondrial and Proteasomal Cross-Talk in PDT

Evidence in cell culture systems showed a relationship between mitochondrial dysfunction and proteasomal impairment. In particular, proteasomal function is affected by the reduction of mitochondrial-dependent ATP synthesis [17]. It was claimed that mitochondrial failure is the primary event followed by proteasome impairment and malfunctioning. The overall effect is the accumulation and aggregation of proteins that further reduce the proteasomal activity, creating a vicious cycle [17].

**Figure 2:**
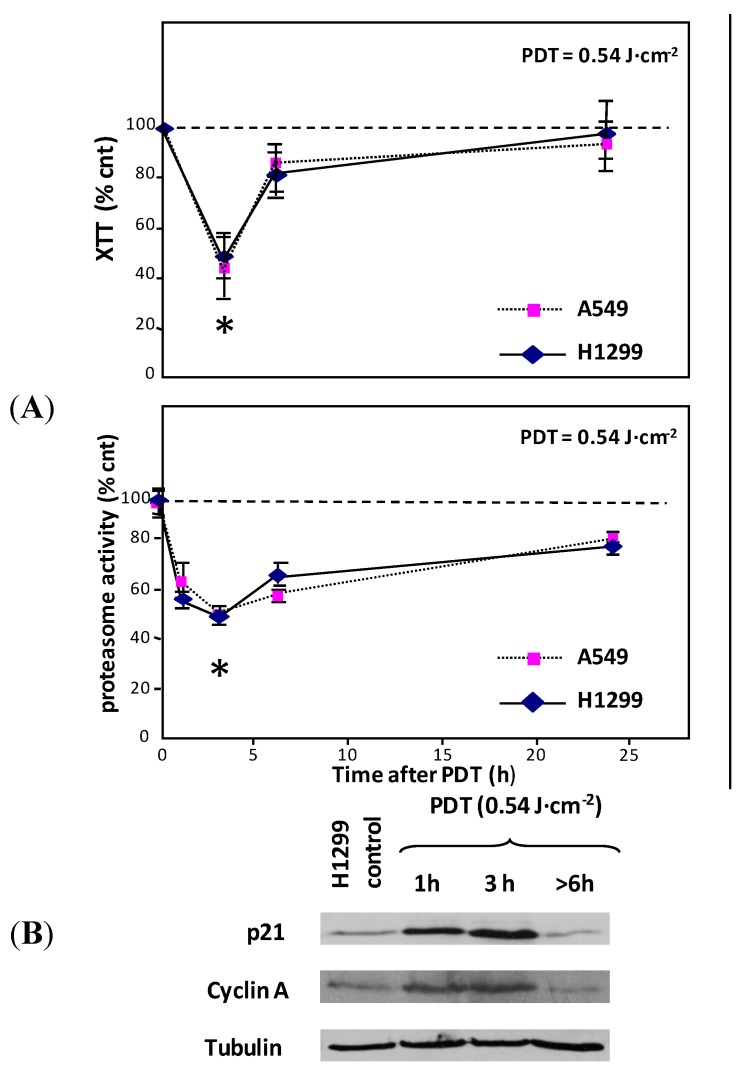
(**A**) Upper and lower panels depict the time-dependent changes of mitochondrial (**upper**) and proteasomal (**lower**) activities after PDT (Photofrin 2.5 μg/mL for 16 h, light fluence kept constant at 0.54 J·cm^−2^). Untreated cells were used as controls corresponding to 100% activity. * *p* < 0.05; (**B**) Expression profiles of p21 and Cyclin A in H1299 cells at 1, 3, and 8 h after PDT (Photofrin 2.5 μg/mL for 16 h, light fluence 0.54 J·cm^−2^). Tubulin was used as loading control.

In this regard, as the oxidative stress by sub-lethal PDT induces a transient dysfunction in mitochondria [18], we would expect negative effects on the 26S proteasome [19]. If this is true, as a consequence of the proteasome stall, the degradation of various proteins would decrease with a parallel accumulation of oxidized proteins. In addition, since p21 is a general genome stress damage sensor, the stressful conditions promoted by PDT contribute to sensibly enhance the level of this cell cycle checkpoint controller (p21). To validate this hypothesis, we evaluated both the general reductive capability of the cell (essentially mitochondrial reductive enzymatic activity) and proteasomal activities. The mitochondrial enzymatic activity was assessed by the XTT assay at 3, 6, and 24 h after photodynamic treatment. Figure 2A shows that the mitochondrial activity of both cell lines appears to decrease in a time-dependent manner. A similar pattern is obtained by assessing the time-dependent changes of chymotrypsin-like activity in H1299 and A549 cells, measured after the same treatment.

Interestingly enough, we observed in both the A549 and H1299 lines that both the mitochondrial and proteasome activities reached their functional minimum exactly at 3 h past PDT and appeared to be decreased to about the same extent (between 50% and 60%). Nevertheless, within 24 h the mitochondrial and proteasomal functions appeared to be significantly recovered.

These observations on the time-dependent decline/recovery of proteasomal activity, determined by sub-lethal PDT, have been fully confirmed by analyzing the expression profiles of some proteins which are known to undergo proteasomal degradation [20]. The profiles of p21 and Cyclin A are shown in the Figure 2B (H1299 cells only). It appears that the expression levels of both proteins first increased within the three hours after irradiation and then decreased progressively, reaching the basal level at ~6 h. Therefore, both direct chymotrypsin-like activity assay and protein profiling show that in our conditions, Photofrin/PDT is responsible for the induction of a significant, albeit transient, stall in proteasome activity.

Additional proof of mitochondria-proteasome cross-talk was obtained when we tried to interfere with the mitochondrial-specific activity using inhibitors of the respiratory chain. In this regard, it has been demonstrated that Antimycin A, one of the first-known and most potent inhibitors of the mitochondrial respiratory chain, binds to the quinone reduction site and blocks Mitochondria Complex I, causing the collapse of the proton gradient across the mitochondrial inner membrane and the inhibition of mitochondrial electron transport [21,22,23]. Similarly, another molecule, namely Rotenone (1-methyl-4-phenylpyridinium), inhibits the transfer of electrons from iron-sulfur centers in Mitochondrial Complex I to ubiquinone and interferes with the respiratory chain, inhibiting NADH oxidation to NAD^+^ [24].

**Figure 3:**
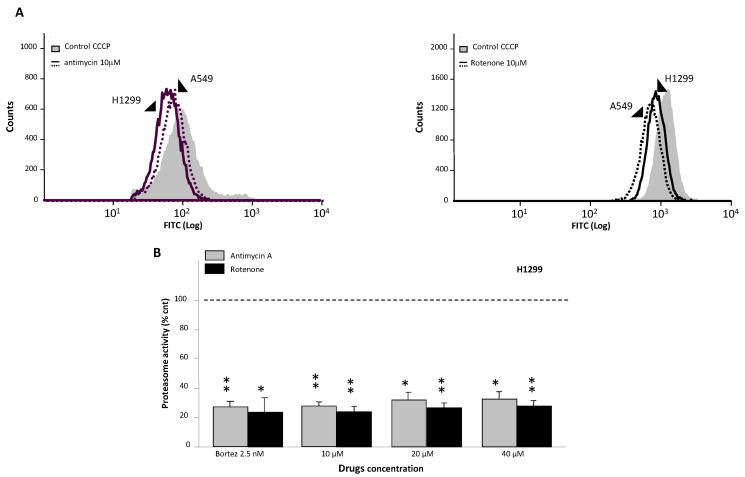
(**A**) ΔΨm decline measured by cytofluorimetric analysis. The fluorescence shift of the MitoPTm TMRE dye in the presence of 10 μM Antimycin (**left**) and Rotenone (**right**) for 3 h; (**B**) Chymotrypsin-like activity as a function of respiratory chain inhibitors Antimycin and Rotenone. Untreated cells were used as controls, while Bortezomib was used as a positive control. * *p* < 0.05; ** *p* < 0.01.

We demonstrated that ΔΨm decreased in H1299 and A549 cells treated for three hours with both electron chain inhibitors (Figure 3A). When ΔΨm collapses, the MitoPTm TMRE potentiometric dye no longer accumulates inside the mitochondria and becomes more evenly distributed throughout the cytosol; consequently, the overall cellular fluorescence drops. The suppression of transmembrane mitochondrial potential is represented by a shift of the fluorescence peak to lower levels. The percentage of H1299 and A549 having this lower fluorescence state amounts respectively to 40% and 48% of the control at the minimal used concentration of Antimycin A (10 μM, 3 h) and Rotenone (10 μM, 3 h) (Figure 3A). The percentage of reduction is similar to that observed in the presence of carbonylcyanide m-chlorophenylhydrazone (CCCP), a well-known chemical inhibitor of oxidative phosphorylation, used as a positive control (not shown).

To further investigate the effects of Antimycin A and Rotenone on proteasome function, we assessed the chymotrypsin-like activity in cells under investigation by incubating them with inhibitors for 3 h. Figure 3B shows that proteasome activity of H1299 was decreased from 65% to 80% independently from the Antimycin A and Rotenone concentrations used (10–40 μM). Comparing the effects of both inhibitors with that of proteasome-specific inhibitor Bortezomib (2.5 nM, positive control), we observed essentially the same efficiency. The same results were obtained also with A549 cell lines (not shown). It is important to underline that concentrations of drugs used in this work spanned from 10 to 40 μM for both inhibitors. Such doses were equal or even lower than those reported by others to inhibit the respective complexes in various cell lines [25].

The data collected indicate that PDT affects the mitochondrial activity as the oxygen radical-generating Photofrin accumulates within these organelles. At the same time, we have shown that PDT, although indirectly, affects the proteasomal activity. If prolonged proteasome arrest induces cell death by apoptosis [26,27], we can favor this situation by using, in sequence, PDT followed by low doses of proteasome inhibitors (or *vice versa*). PDT, beside its direct killing properties, is able to induce proteasome arrest. The combination of PDT with a specific proteasome inhibitor should result in amplified therapeutic efficiency. At this stage, then, we considered it important to study the effects of Bortezomib alone or in combination with PDT on our cells.

### 2.4. Bortezomib Induces a Transient Proteasome Arrest in H1299 and A549 Cell Lines

Bortezomib (BZT) is a drug approved in 2003 by the US Food and Drug Administration for the treatment of patients with relapsed or refractory multiple myeloma [28,29]. At molecular level, Bortezomib selectively binds to a threonine hydroxyl group in the chymotrypsin-like active site of proteasome, blocking its activity [29].

Although clinically employed specifically for the cure of multiple myeloma, in recent years a great number of clinical studies have been conducted to evaluate Bortezomib’s therapeutic potential in many neoplasias including renal, prostate, and lung cancers [30,31].

Despite the large number of published or ongoing investigations, the molecular mechanisms by which Bortezomib elicits responses in cells are still the subject of investigations. Some recent evidence demonstrated, however, that treatment of non-small-cell lung cancer cells (H460) with Bortezomib caused Gap2/Mitosis (G_2_/M) phase arrest [32]. This block was associated with increased phosphorylation and cleavage of the anti-apoptotic protein Bcl-2 and the accumulation of cyclins A and B [33]. Another described effect of Bortezomib involves the deregulation of NF-κB activity, since, as a consequence of the drug-mediated proteasome arrest, its inhibitor IκBα cannot be degraded and the transcription factor does not translocate to the nucleus. This situation may be even more complicated since NF-κB rules other activities such as cytokine-mediated proliferation, angiogenesis, and development of tumor metastasis [33]. In many cases, Bortezomib treatment effected the enhanced expressions of the main stress sensor p53 and p21, and significant reactive oxygen species production and apoptosis [32,34].

**Figure 4:**
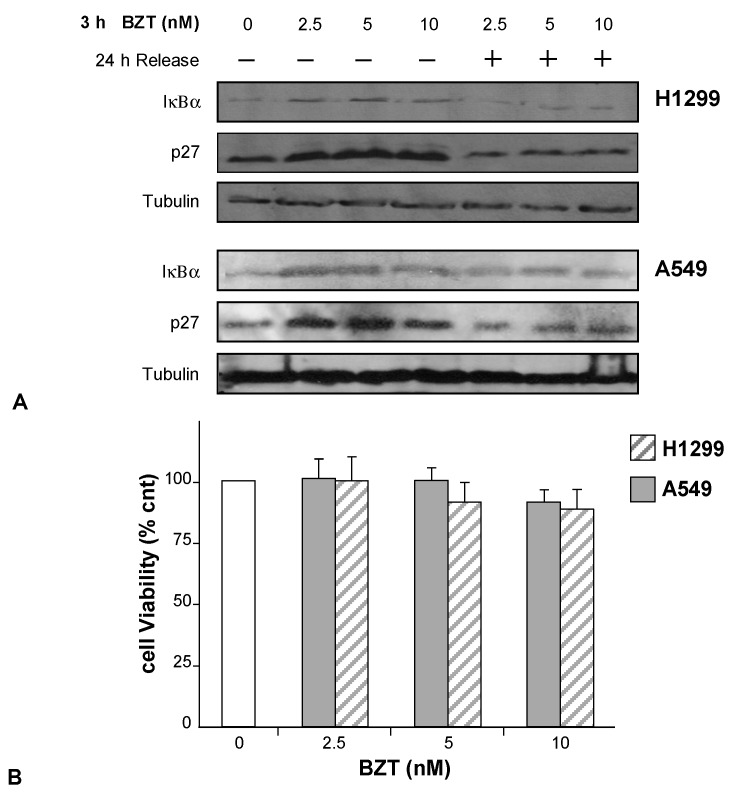

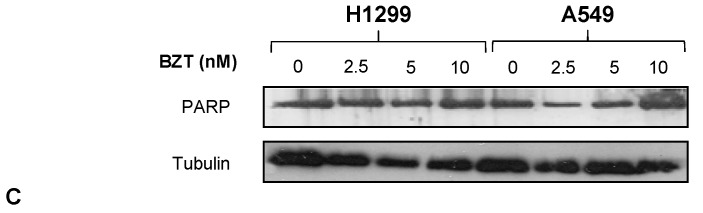
(**A**) Expression profiles of p27 and IκBα in H1299 and A549 cells incubated with Bortezomib (BZT) at the indicated concentrations (lanes 2–4) and after 24 h of release in fresh medium (lanes 5–7). Tubulin was used as a loading control; (**B**) Reversible effects of Bortezomib at various concentrations (3 h incubation) on cell viability of H1299 and A549 cells (XTT assay); the analyses were performed after 24 h of release in fresh medium; (**C**) Expression profile of PARP (poly ADP-ribose polymerase) in H1299 (lanes 1–4) and A549 cells (lanes 5–8) incubated with BZT at the indicated concentrations. Tubulin was used as gel loading control.

Among the numerous *in vitro* studies that concern the effect of Bortezomib on cell lines, the screening performed in the 1990s on a panel of 60 cell lines by the National Cancer Institute is particularly important. It showed that Bortezomib potently inhibited the growth of all cells at a concentration around 7–10 nM [32,35,36]. Keeping in mind the use of Bortezomib in combination with PDT, we first decided to analyze the effects of the drug alone in H1299 and A549 lung adenocarcinoma cell lines. For this purpose, we exposed these cells to Bortezomib (2.5, 5.0, and 10 nM) for 3 h and estimated p27, a cyclin dependent kinase (Cdk) inhibitor protein, and IκBα expression levels in both cell lines by Western blot. As shown in Figure 4A (lanes 2–4), the expression of these proteins increased in both H1299 and A549 cells within the first 3 h at all employed concentrations, suggesting a significant inhibition of proteasome activity. The hypothesized arrest of proteasome in these conditions has been confirmed by direct chymotrypsin-like activity quantitative assay (not shown). In these conditions, however, we could not demonstrate any sign of cytotoxicity as assessed by XTT assay and, more importantly, we could not demonstrate any sign of apoptosis as suggested by the absence of PARP (poly ADP-ribose polymerase) cleavage (Figure 4B,C).

To check whether, in our conditions, *i.e.*, low Bortezomib concentrations and 3 h of incubation, the block of proteasome activity was reversible or not, we released treated cells in fresh complete medium for 24 h and analyzed the expression levels of p27 and IκBα proteins. As shown in Figure 4A (lanes 5–7) the levels of both p27 and IκBα proteins were declining after the release in a Bortezomib-free medium.

### 2.5. Combined Therapy

At present, PDT is a stand-alone therapy for select tumors [1,37]. However, *in vitro* studies have shown that PDT combined with other therapeutic measures might enhance tumor cell death [38]. Hence, we have compared the molecular effects on both cell lines of individual treatments (PDT or Bortezomib) *versus* a combined PDT and Bortezomib treatment.

Recently, it has been shown that the combination of Bortezomib (and also other proteasome inhibitors) with a chemotherapeutic agent may increase the overall anti-tumor efficacy in both *in vitro* and *in vivo* models. In this regard, it has been demonstrated that the use of Irinotecan or Gemcitabine combined with Bortezomib in pancreatic and colon cancer xenograft models determined tumor regression, although neither Irinotecan, Gemcitabine, nor Bortezomib had striking activity as single agents for these typically refractory cancers [39,40]. Similarly, Bortezomib in combination with 5-fluorouracil, cisplatin, docetaxel, or doxorubicin produced additive anti-tumor and anti-metastatic effects in different xenograft models including lung cancer [39]. Russo *et al.* showed that Bortezomib increases radiation-induced apoptosis and radiosensitivity in colorectal cancer cells *in vitro* and *in vivo* [41].

The observations that we have reported, while confirming the ability of low Bortezomib concentrations to transiently block the proteasome, indicate that PDT may also rapidly, albeit transiently, have the same capability. If these properties could be confirmed, it can be assumed that a combination of the two therapies, resulting in a prolonged proteasome arrest, could bring the surviving cells to die. Indeed, this assumption is sustained from various reports stating that prolonging the stall of proteasome activity would bring tumor cells to apoptosis [40].

**Figure 5:**
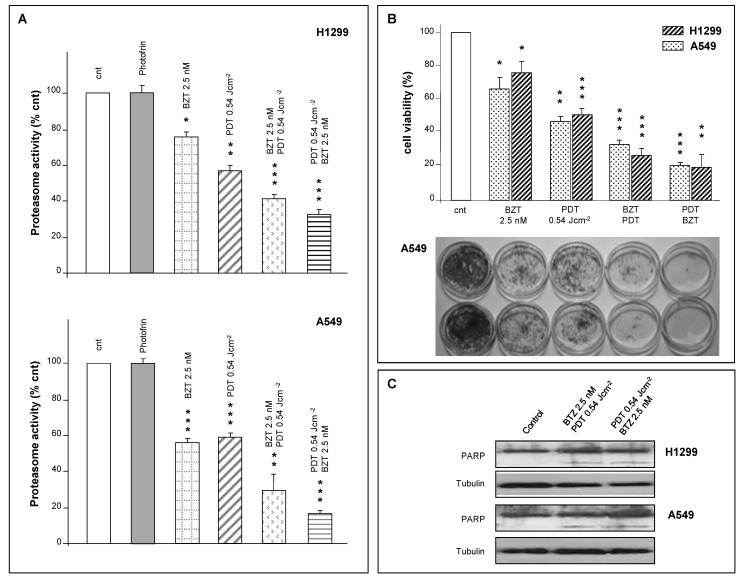
(**A**) Proteasome activity in H1299 and A549 cells under single or combined treatments. * *p* < 0.05; ** *p* < 0.01; *** *p* < 0.001; (**B**) **Upper panel**: Cell viability of both cell lines measured by XTT assay 24 h after individual or combined treatments. * *p* < 0.05; ** *p* < 0.01; *** *p* < 0.001; **Lower panel**: Colony-forming assay (A549 cells, only) after >7 days from individual or combined treatments; (**C**) Expression profile of PARP in H1299 and A549 cells under combined therapy regimens. Tubulin was used as loading control.

To evaluate the potential therapeutic effects in combination, we analyzed the proteasome activity of cells subjected to combined treatments. Light fluence was 0.54 J·cm^−2^, while Bortezomib was kept at 2.5 nM (3 h incubation). Given that preclinical studies have indicated that the sequence of administration in the combination regimen is decisive for the best result, we also analyzed the effects of combination treatments either administering Bortezomib to cells pre-subjected to PDT or *vice versa*. We obtained a very significant decrease in chymotrypsin-like activity in both cell lines which spanned from about 65% up to 85% (Figure 5A, upper and lower panels). In this regard, we observed that the effect on the chymotrypsin-like activity of both cell lines is irrespective of whether PDT or Bortezomib is administered first, *i.e.*, PDT followed by Bortezomib incubation or Bortezomib incubation followed by PDT. Although observed *in vitro*, this effect seems very important since literature data were restricted to a single and extreme modality of administration [42]. At variance, our data also indicate that PDT treatment followed by Bortezomib can be even more efficient, despite milder conditions being used.

Our experiments show also that effects caused by single or combined treatments were more important in A549 (p53 positive cells) compared with those observed in H1299 (p53 null cells). In this regard, our data are in agreement with those of Strauss *et al.* [37], who showed that Bortezomib cytotoxicity may depend on the sensitivity of a specific cell line.

We have also evaluated the effects of individual and combined treatments at 24 h after treatment by XTT assay and at much longer time intervals (7–8 days) by colony-forming assay. In any case, we found that the viability of H1299 and A549 cells subjected to combined treatment and their efficiency in forming new colonies was dramatically reduced when compared to individual treatments (Figure 5B). It is important to underline that the inhibition of the proliferative power of both cell lines is also held at much longer times, as demonstrated by the colony-forming assay (performed after >7 days).

We have shown that both PDT and Bortezomib at the indicated conditions were able to induce a transient stall in the proteasome activity of H1299 and A549 cells. The mechanism of proteasome inhibition by PDT remains partially elusive, but the reduction of mitochondrial activity may be responsible for it. In our stand-alone conditions, the proteasome arrest is transient and does not involve the activation of an apoptotic program. This may not be the case of a combination treatment if, according to some authors, a protracted stall of proteasome activity may, indeed, trigger apoptosis [20,26,27]. To validate this hypothesis we have analyzed PARP cleavage by Western blotting.

Indeed, combined treatment results in a clear PARP cleavage. This cleavage, in addition, is independent of the order of administration, suggesting that under both regimens of combined therapy, H1299 or A549 cells activate an apoptotic program (Figure 5C).

## 3. Experimental Section

### 3.1. Cells Culture

The H1299 human non-small-cell lung cancer cell line (p53^−/−^) was obtained from American Type Culture Collection (Rockville, MD, USA) and grown in RPMI 1640 (Roswell Park Memorial Institute medium), 2 mM l-glutamine, 10 mM HEPES (4-(2-hydroxyethyl)-1-piperazineethanesulfonic acid), 1 mM sodium pyruvate, 4500 mg/L glucose, 1500 mg/L sodium bicarbonate, 100 μg/mL streptomycin, 100 units/ml penicillin, and 10% FCS (fetal calf serum).

The A549 human non-small-cell lung cancer cell line (p53^+/+^) was obtained from American Type Culture Collection and grown in Ham’s F12K, 2 mM l-glutamine, 1.5 mg/L sodium bicarbonate, 100 units/ml penicillin, and 10% FCS. All media and cell culture reagents were purchased from Gibco (Invitrogen, Waltham, MA, USA).

### 3.2. Reagents

Photosensitizer: The Photofrin (hematoporphyrin derivative Porfimer sodium) used in this work was supplied as freeze-dried powder (Axcan Pharma, Mont-Saint-Hilaire, QC, Canada). A Photofrin stock solution was prepared by dissolving the powder in water containing 5% glucose to obtain a final concentration of 2.5 mg/mL (stored at −20 °C in the dark). In all PDT experiments, cells were incubated at 37 °C in the dark with 2.5 μg/mL of Photofrin for 16 h before irradiation.

Bortezomib: The proteasome inhibitor Bortezomib was obtained from Millennium Pharmaceuticals Inc. (Cambridge, MA, USA). This compound was dissolved in DMSO (dimethyl sulfoxide) to obtain 1 μM stock solution.

Antimycin A: The respiratory chain inhibitor Antimycin A was obtained by Sigma Aldrich (St. Louis, MO, USA). Stock solution (1 mM) was prepared by directly dissolving the powder in ethanol. Antimycin A acts by inhibiting the oxidation of ubiquinol in the electron transport chain of oxidative phosphorylation.

Rotenone: The Mitochondrial Complex I inhibitor Rotenone was obtained by Sigma Aldrich (St. Louis, MO, USA). This compound was dissolved in chloroform to obtain a stock solution of 25 mM.

### 3.3. Subcellular Localization of Photofrin

Localization of Photofrin has been ascertained by direct microscope observation of the fluorescence emitted by the cells incubated with the photosensitizer while growing on cover slips. In particular, about 2.4 × 10^4^ H1299 cells were seeded into p35 plates for 24 h and incubated with Photofrin (2.5 µg/mL for 16 h) in the dark and then washed with Hank’s solution.

The overall mitochondrial system was visualized by staining cells with 50 mM MitoTracker Green FM (Invitrogen) for 30 min at 37 °C (the excitation wavelength at 490 nm and the emission wavelength at 516 nm). Images were captured using a Zeiss LSM 510 Meta argon/krypton laser-scanning confocal microscope (Carl Zeiss Microscopy GmbH, Jena, Germany). Four images from each optical section were averaged to improve the signal-to-noise ratio.

### 3.4. Mitochondrial Membrane Potential (ΔΨm) Assessment

About 4 × 10^5^ cells were incubated with concentrations of Antimycin and Rotenone from 0 to 40 µM. After 3 h, the mitochondrial membrane potential (ΔΨm) was performed using MitoPTm TMRE (Immunochemistry Technologies, LLC-Limited Liability Company, Bloomington, MN, USA). The assay is based on a slow, lipophilic, cationic fluorescent redistribution of tetramethylrhodamine ethyl ester (TMRE). Cells labeled with MitoPTm TMRE were analyzed in triplicate by flow cytometer using a CyAn ADP Flow Cytometer (Beckman Coulter, Brea, CA, USA) and Summit Software (Beckman Coulter, Brea, CA, USA).

### 3.5. Mitochondrial Activity and Cell Viability Assay

Cell viability was assayed on about 5 × 10^4^ cells in triplicate into 96-well plates using Cell Proliferation Kit II (XTT) (Roche, Penzberg, Upper Bavaria, Germany). The assay is based on the cleavage, possible only in viable cells, of the yellow tetrazolium salt XTT to form an orange formazan dye with metabolic active cells. The increase in the overall activity of mitochondrial dehydrogenases of samples correlates with the amount of dye released (measurable spectrophotometrically at 490 nm) and is proportional to the effective number of live cells.

Optical densities at 490 nm were collected with an ELISA plate reader (Bio-Rad, Hercules, CA, USA). Data are expressed as percentage of treated cells *versus* untreated controls (mean ± S.D.).

### 3.6. Individual Treatments

PDT: Cells were irradiated by a broadband light delivered with a PTL-Penta apparatus (Teclas, Sorengo, Switzerland). This apparatus consists of a halogen lamp (Osram, 250 W, 24 V, Munich, Germany) equipped with a bandpass filter (>80% transmittance in the 510 to 590 nm spectral region; bandwidth ~40 nm at 50% of the peak) corresponding approximately to one of the Photofrin absorption peaks. The emission spectrum was measured with a MacaSR9910 spectroradiometer (Macam Photometrics, Livingston, Scotland, UK). Light was delivered through an 8-mm bundle of optical fibres placed at a distance from cell plates that ensured uniform illumination of the entire cell monolayer. Fluence rate at level of the cell monolayer was fixed at 6 mW·cm^−2^ and light was irradiated at the required doses (J·cm^−2^). Photofrin concentration was set at 2.5 μg/mL and light dose at 0.54 ± 0.02 J·cm^−2^. The selection of this specific irradiation protocol originates from a previous report [40]. Using these conditions, the reduction of cell viability in the dark was small, amounting to about 2%–3%. In turn, exposing cells to a light fluence of 0.54 ± 0.02 J·cm^−2^, mortality was about 60%. Both cells lines were fully insensitive to light fluences up to 1.8 J·cm^−2^, in the absence of the photosensitizer.

Bortezomib: According to our previous work on the same cells [17], these were incubated for 3 h with concentrations of Bortezomib from 0 to 10 nM and analyzed immediately or after a 24-hour release in fresh medium.

### 3.7. Combined Treatments

Combination experiments were performed by keeping constant light fluence and Bortezomib concentration, *i.e.*, 0.54 J·cm^−2^ and 2.5 nM, respectively.

Two modalities of administration were adopted:

(a) Administration of Bortezomib followed by PDT treatment. H1299 and A549 cell lines were treated with Bortezomib for 3 h, then incubated with Photofrin (16 h), and irradiated. Cells were analyzed 24 h later.

(b) PDT treatment followed by Bortezomib administration. Cells incubated with Photofrin (16 h) were irradiated, rinsed, and treated for additional 3 h with Bortezomib. Cells were analyzed 24 h later.

### 3.8. Colony-Forming Assay

Colony-forming efficiency was assayed in duplicates by seeding ~2 × 10^3^ cells in 35 mm plates. Twenty-four hours later, cells were exposed to both single and combined treatments. After ≥7 days culture, colonies (>50 cells) were stained with 1% methylene blue in 50% ethanol for two hours and photographed (Nikon, Shinagawa, Tokyo, Japan).

### 3.9. Western Blot Analysis

Total cell protein preparations were obtained by lysing cells in 50 mM Tris (pH 7.5), 100 mM NaCl, 1% NP40, 0.1% Triton, 2 mM EDTA, 10 μg/mL aprotinin, and 100 μg/mL phenylmethylsulfonyl fluoride. Protein concentration was routinely measured with the Bio-Rad protein assay [43]. Polyacrylamide gels (10%–15%) were prepared essentially as described by Laemmli [44]. Proteins separated on polyacrylamide gels were blotted on to nitrocellulose filters (Perkin Elmer, Waltham, MA, USA). Molecular weight standards were from Fermentas Life Sciences (M-Medical, Milan, Italy). Filters were stained with specific primary antibodies and then with secondary antisera conjugated with horseradish peroxidase (Bio-Rad, Hercules, CA, USA). Filters were developed using an electro-chemiluminescent Western blotting detection reagent (Roche); profiles were acquired and quantified by scanning with a Discover Pharmacia scanner equipped with a Sun Spark Classic Workstation. The anti-p27, anti-IκBα, anti-cyclin A, anti-PARP, and anti-p21 antibodies were from Santa Cruz Biotechnology (Santa Cruz, CA, USA). Anti-tubulin (MCA77G) was from Serotec (Kidlington, UK).

### 3.10. Proteasome Activity Assay

Proteasome activity assay was tested using Proteasome-Glo™ Chymotrypsin-Like, Trypsin-Like, and Caspase-Like Cell-Based Assays (Promega, Fitchburg, WI, USA). This assay monitors the chymotrypsin-like, the trypsin-like, and the post-glutamyl peptide hydrolytic or caspase-like activities, which are the main enzymatic constituents of the 20S core of proteasome.

About 7.0 × 10^3^ cells were seeded into 96-well plates for 24 h. These cells were then exposed to individual or combined therapeutic regimens as outlined above.

The assay was performed in strict accordance to manufacturer’s instructions. To this purpose, cells were equilibrated to room temperature and treated with the Proteasome-Glo™ Cell-Based Reagent and culture medium 1:1 for 5 min with continuous shaking (~350 rpm). Luminescence signals of triplicate samples were detected using a Glomax microplate luminometer (Promega). The well-known proteasome inhibitor Bortezomib was used as positive control (2.5 nM). Results, expressed as percent of the activity present in control cells, are reported as mean ± S.D.

### 3.11. Statistical Analysis

Significance was assessed by the Student’s *t*-test for unpaired, independent data for comparisons between two means. Each experimental point has tested against the relative untreated control. *p* values: * *p* < 0.05; ** *p* < 0.01; *** *p* < 0.001.

## 4. Conclusions

In conclusion, our data paradigmatically suggest that the inhibition of proteasome activity using combination treatment may be an additional strategy to enhance the death of apoptosis-resistant cancer cells. Combination may offer several advantages, including the possibility of enhancing and exalting general therapeutic effects from additivity up to synergy. This implies that toxic drugs may be possibly used at lower concentrations, thus minimizing detrimental side effects. The combination of PDT, a side effect-void therapy (except for a prolonged photosensitivity), with Bortezomib appears particularly convenient and effective considering that it can be used at concentrations so low that it induces only a transient proteasome arrest when used alone.
